# Case report: Long-term voluntary Tyrosine Kinase Inhibitor (TKI) discontinuation in chronic myeloid leukemia (CML): Molecular evidence of an immune surveillance

**DOI:** 10.3389/fonc.2023.1117781

**Published:** 2023-03-16

**Authors:** Jusuf Imeri, Christophe Desterke, Paul Marcoux, Diana Chaker, Noufissa Oudrhiri, Xavier Fund, Jamila Faivre, Annelise Bennaceur-Griscelli, Ali G. Turhan

**Affiliations:** ^1^ INSERM Unité Mixte de Recherche (UMR)_S_1310, Université Paris Saclay, Villejuif, France; ^2^ INGESTEM National IPSC Infrastructure, Villejuif, France; ^3^ CITHERA, Center for iPSC Therapies, Evry, France; ^4^ APHP Paris Saclay, Division of Hematology, Paris Saclay University Hospitals, Le Kremlin Bicêtre, and Villejuif, France; ^5^ Inserm Unité Mixte de Recherche (UMR) 1193 Centre-Hepato Biliaire, Paul Brousse, Villejuif, France

**Keywords:** chronic myelogenous leukemia, natural killer cells, NKG7, TFR, CML

## Abstract

The classical natural history of chronic myeloid leukemia (CML) has been drastically modified by the introduction of tyrosine kinase inhibitor (TKI) therapies. TKI discontinuation is currently possible in patients in deep molecular responses, using strict recommendations of molecular follow-up due to risk of molecular relapse, especially during the first 6 months. We report here the case of a patient who voluntarily interrupted her TKI therapy. She remained in deep molecular remission (MR4) for 18 months followed by detection of a molecular relapse at +20 months. Despite this relapse, she declined therapy until the occurrence of the hematological relapse (+ 4 years and 10 months). Retrospective sequential transcriptome experiments and a single-cell transcriptome RNA-seq analysis were performed. They revealed a molecular network focusing on several genes involved in both activation and inhibition of NK-T cell activity. Interestingly, the single-cell transcriptome analysis showed the presence of cells expressing NKG7, a gene involved in granule exocytosis and highly involved in anti-tumor immunity. Single cells expressing as granzyme H, cathepsin-W, and granulysin were also identified. The study of this case suggests that CML was controlled for a long period of time, potentially *via* an immune surveillance phenomenon. The role of NKG7 expression in the occurrence of treatment-free remissions (TFR) should be evaluated in future studies.

## Introduction

Chronic myeloid leukemia (CML) is a clonal myeloproliferative neoplasm initiated by the occurrence of the Philadelphia chromosome (Ph1) in a primitive hematopoietic stem cell. The natural history of disease from a chronic phase towards accelerated and blast phases has now been drastically modified by the introduction of the targeted therapies. BCR::ABL1 tyrosine kinase inhibitors (TKIs) improved overall survival in patients with chronic phase CML (1), and recent data suggest that the life expectancy of CML patients responding to TKI could currently be similar to that of the general population of the same age and sex (2).

Up to 80% of newly diagnosed CML patients in chronic phase (CP) achieve a molecular response under TKI therapies especially by the use of second-generation TKI such as Nilotinib (3). A major issue remaining today in the CML field is the persistence of stem cells in deep molecular response due to the resistance of leukemic stem cells (LSC) to TKI (4–6). Indeed, TKI treatment is unable to eliminate quiescent stem cell fraction (7), and the survival of LSC is independent on BCR::ABL TK activity (8). In half of the CML patients in deep molecular responses, TKI discontinuation can allow a treatment-free remission (TFR) (9, 10). In several TKI-discontinuation trials, it has been shown that an immunological mechanism contributes to the absence of relapse, in particular with increased levels of NK cells (11).

We report here the case of a patient who voluntarily stopped her TKI therapy and remained in deep molecular response without therapy for 18 months. At the end of this period of 18 months, which was operationally a TFR, a molecular relapse was diagnosed, but the patient declined therapy for an additional 3 years until hematological relapse. A transcriptome analysis was performed at several time points of this TKI-discontinuation condition with low or high BCR::ABL^IS^ levels. In addition, a single-cell transcriptome analysis of the circulating mononuclear cells was performed.

## Case description

**Figure 1:**
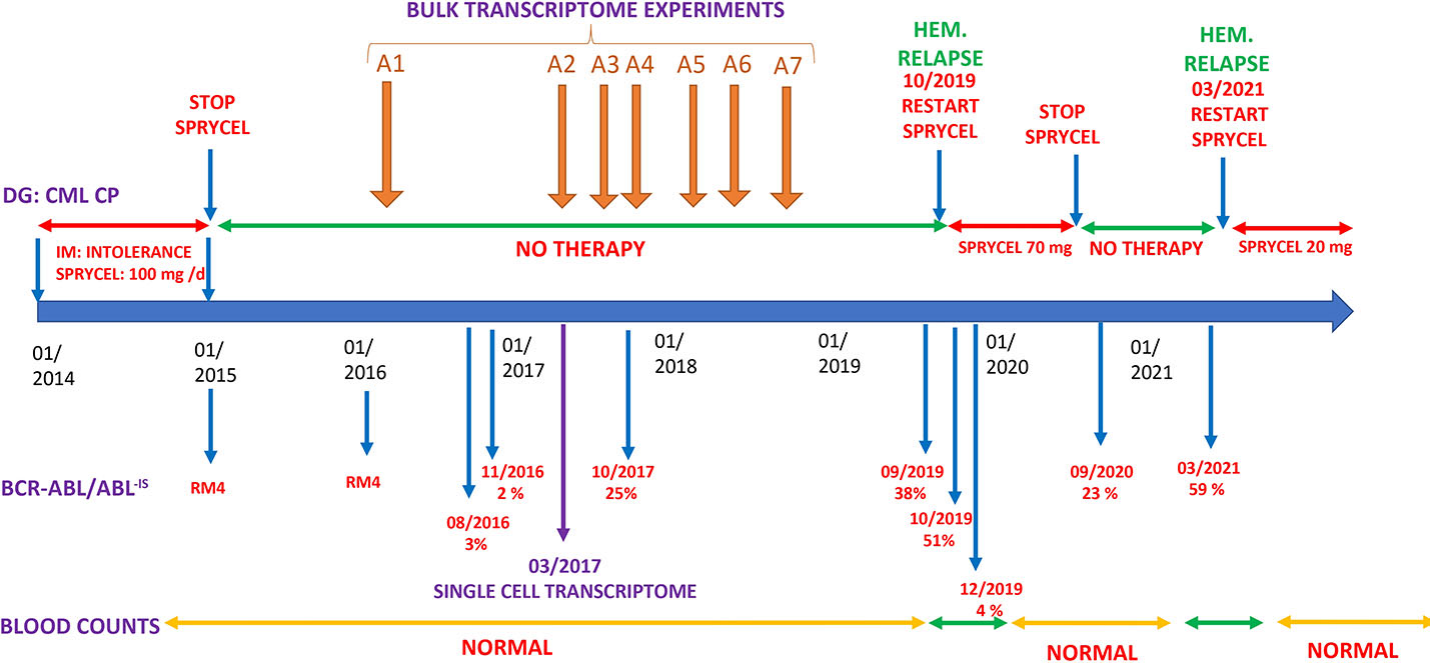
Clinical history. DG, diagnosis; IM, imatinib; Hem. relapse, hematological relapse (see text).

A CML was diagnosed in 2014 in a 30-year-old female patient with low Sokal score. She was initially treated with Imatinib, but this drug was rapidly stopped because of intolerance, and she was switched to Dasatinib (**Figure 1**). A deep molecular response (RM4) was rapidly obtained with a clinical and molecular follow-up every 3 months. At January 2016, she stopped her therapy voluntarily. In August 2016, molecular analysis identified a relapse with BCR::ABL/ABL^IS^ levels, which rose to 3%. She disclosed at that time that she was not taking Dasatinib. Despite this molecular recurrence, she declined therapy while accepting regular hematological and molecular analyses. BCR::ABL^IS^ levels rose to 25% in October 2017 and to 38% in September 2019 (**Figure 1**). During this 3-year period of molecular progression, blood counts and clinical examinations remained normal. In October 2019, BCR::ABL^IS^ was 51%, and she was in hematological relapse. She refused bone marrow aspirate but accepted to start TKI therapy by Dasatinib 100 mg/day. After an initial response, she reduced the dose of Dasatinib to 50 mg/day because of GI intolerance and stopped it again in September 2020. In March 2021, she was again in hematological relapse with BCR::ABL^IS^ level at 59%. She accepted Dasatinib at a very low dose (20 mg/day), which induced a major molecular response (MMR) that is currently ongoing.

To gain insights into the molecular events occurring during this “self-induced” drug interruption, we have performed with the informed consent of the patient, serial transcriptome analyses in frozen peripheral blood cells (PBMCs). The first three were performed during periods of low BCR::ABL expression, whereas the last four were performed in samples with high BCR::ABL expression (**Figure 1**). All these analyses were performed without TKI administration. In one sample, a single-cell RNA-seq analysis was performed. For all retrospective samples for which BCR::ABL^IS^ quantification was performed, peripheral blood mononuclear cells have been processed to perform total RNA extraction and whole transcriptome experiment on microarray chip ClariomS human (Thermo Fisher Scientific).

## Discussion

Although this patient declined therapy for a long period of time (5 years), the progression of the disease was very slow. This prompted us to perform bulk transcriptome analyses from serially collected blood samples to study the expression of the genes involved during this natural history (**Figure 1**). For each timepoint of these samples, the results of BCR::ABL^IS^ quantifications were available. BCR::ABL-IS quantification was taken as predictor for Pavlidis Template Matching algorithm (12) to elucidate gene expression profile, which followed the amount of BCR::ABL transcript during the time course of the disease. After false discovery rate adjustment of the gene signature, 188 genes were found to be connected to BCR::ABL^-IS^ (**Supplemental Table S1**). One of the top associated molecule was the BCR transcript (rank = 30), suggesting a good correlation between BCR::ABL^-IS^ quantification and transcriptome time-course experiments.

### Evidence of the enrichment of an immunological signature during the time out of therapy

**Figure 2:**
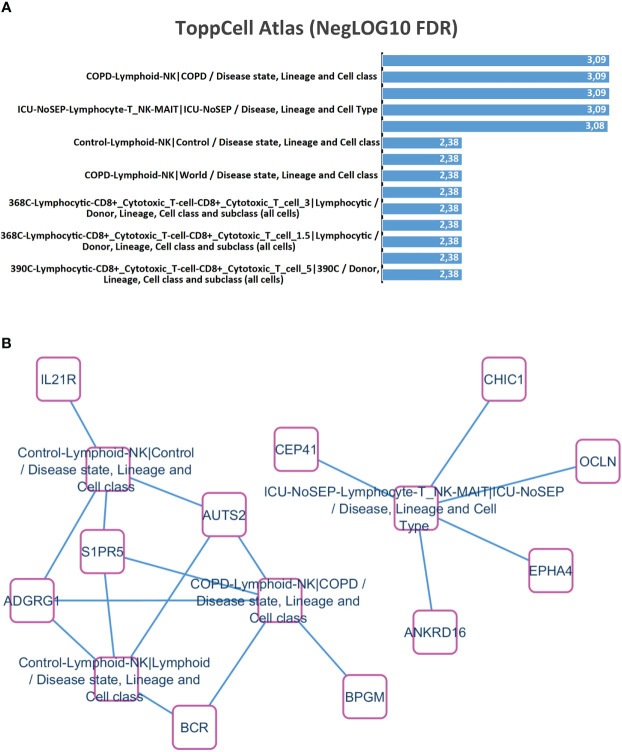
NK/T lymphocyte cell-type enrichment during time course transcriptome experiments correlated with BCR::ABL^-IS^ quantification. **(A)** Barplot of functional enrichment performed on ToppCell Atlas database (negative log10 of false discovery rate p-values); **(B)** NK-T lymphocyte cell enrichment network.

Functional enrichment performed with time course transcriptome expression profile (**Supplemental Table S1**) on ToppCell Atlas database revealed that this signature mainly characterized natural killer-T (NK-T) lymphocyte cell type (**Figure 2A**), with specific upregulation of IL21R, ADGR1, S1PR5, AUTS2, BCR, BPGM, ANKRD16, EPHA4, OCLN, CHIC1, and CEP41 (**Figure 2B**). These results suggested that during the natural evolution of the disease, the molecular signature of an NK/T lymphocyte cell population could be detected in the PBMC.

A single-cell RNA-seq analysis was performed on the PBMC sample collected on March 2017 where the patient had evident molecular progression with a blood BCR::ABL^-IS^ level at 2%. She was out of therapy at that time (**Figure 1**), and her blood counts were normal.

**Figure 3:**
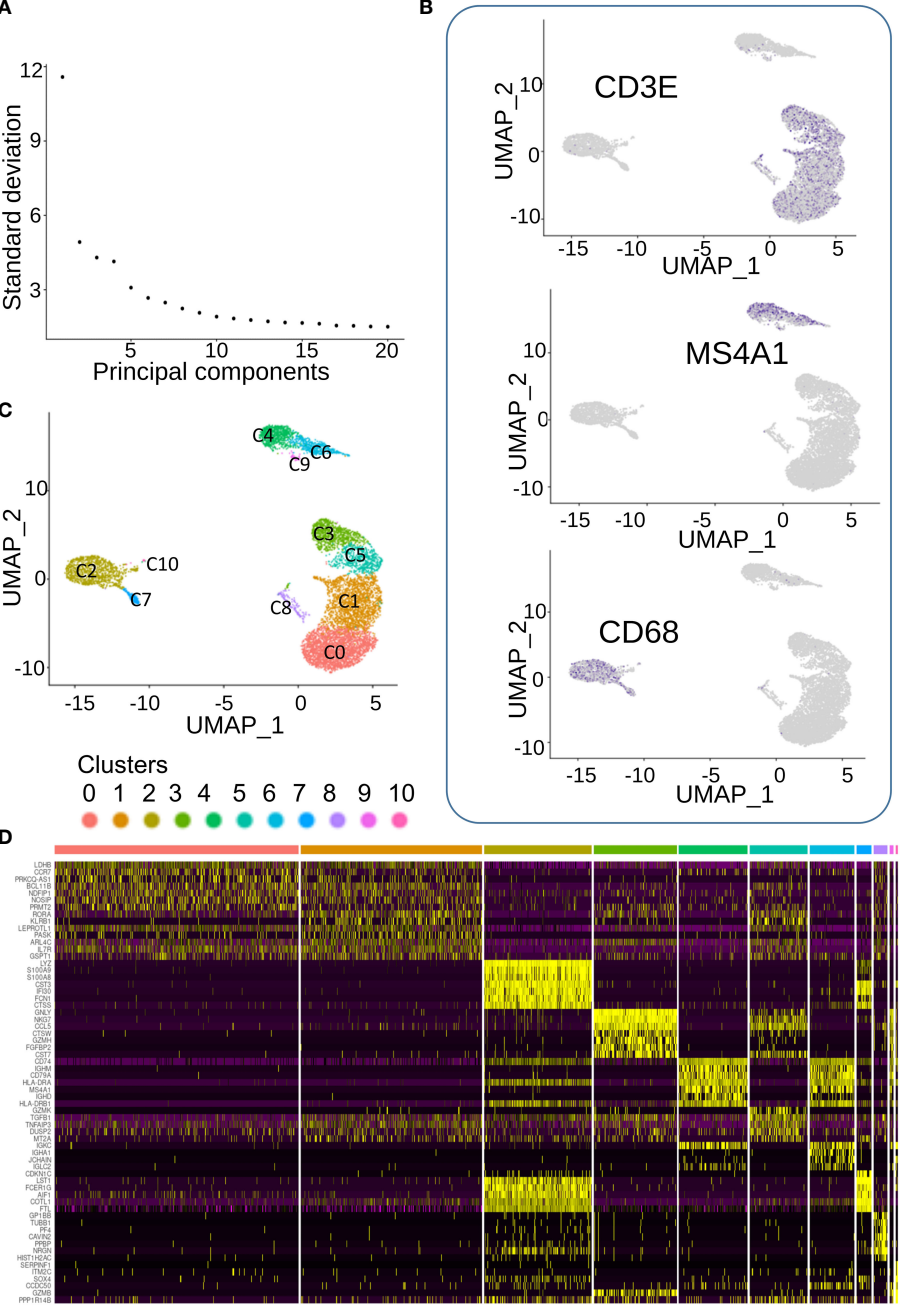
Single cell transcriptome analysis of CML blood cells. **(A)** Elbow plot of dimension reduction. **(B)** Feature plot of markers characterizing three main cell populations: CD3E for T lymphocyte, CD68 for monocytes, and MS4A1 for B lymphocytes. **(C)** Unsupervised clustering on UMAP dimension reduction. **(D)** Expression heatmap of best molecular markers of the eleven cell communities.

Single-cell transcriptome of 9,036 PBMC could be demultiplexed after Cell Ranger pipeline. A mean number of 9,054 transcripts has been found to be expressed by cell. After preprocessing of single-cell transcriptome of PBMCs, dimension reduction by PCA (**Figure 3A**) and UMAP identified three main topological cell clusters including T-lymphocytes (expressing CD3E), B-lymphocytes (expressing MS4A1), and monocytes expressing CD68 (**Figure 3B**). UMAP dimension reduction conjugated to unsupervised clustering identified 11 distinct cell communities (**Figure 3C**), which expressed distinct patterns of markers (**Figure 3D**). Clusters 7–10 were found to be less represented quantitatively in the blood of the patient (**Figure 3D**).

**Figure 4:**
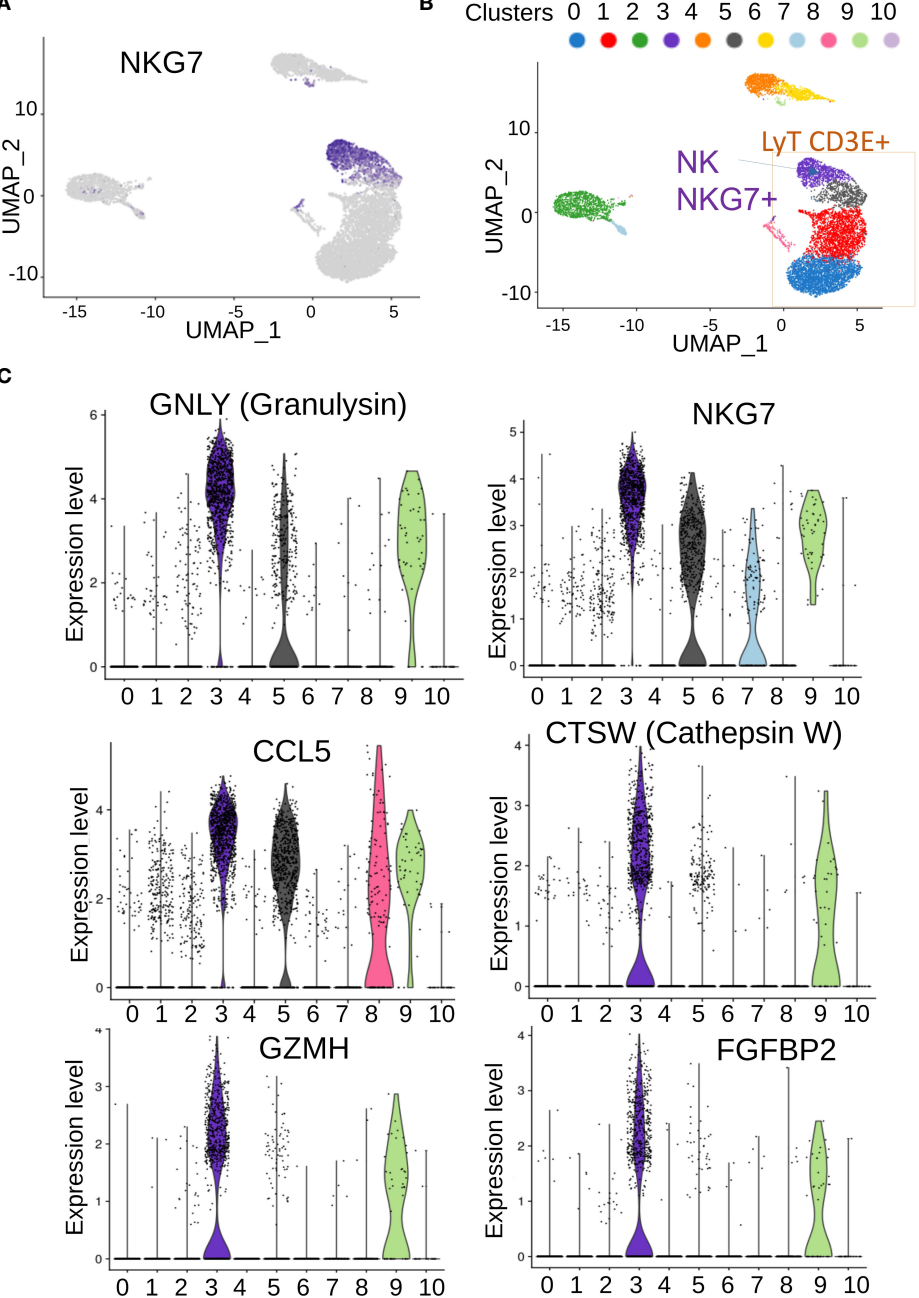
Characterization of genes associated with NK/T cells in PBMC. **(A)** UMAP of NKG7-positive cells in single-cell transcriptome; **(B)** inference of NKG7-positive cell on cells communities identified; **(C)** best over expressed markers identified in cluster 3 of NKG7-positive cells.

Clusters 0 and 1, which were the largest ones, seemed to share expression of markers (**Figure 3D**) corresponding to T lymphocytes (**Figures 3B, C**). In terms of expression specificity, cluster 5 shared some markers with clusters 0 and 1(**Figure 3D**). Interestingly, among the cluster of T-lymphocytes (**Figures 3B, C**), we could observe another cluster (cluster C3) that expressed a distinct signature from the three other T-lymphocyte clusters (0, 1, and 5, **Figure 3D**). Among the best markers characterized during single-cell transcriptome analysis, a high expression of NKG7 was revealed in individual cluster C3 (**Figure 4A**), which correspond to a subpopulation of CD3E+ T lymphocytes (**Figure 4B**). Differential expression analysis between the distinct cell clusters showed that a high expression of natural killer effective markers characterized cluster C3 (**Figure 4C**), such as GNLY (granulysin), GZMH (granzyme H), and CTSW (cathepsin W). In addition, a high expression of CCL5 chemokine and FGFBP2 secreted protein (cytotoxic marker) was found in cluster C3 of natural killer cells from this patient (**Figure 4C**). These molecular results on single-cell transcriptome performed on PBMC suggested therefore the presence of an immunologically competent cells in the blood sample analyzed.

Interestingly, functional enrichment of the gene signature on ToppCell atlas signature highlighted that activation of genes involved in natural killer/T lymphocytes following BCR::ABL quantification. NK cells play important roles in innate immunity against virus or tumors by secreting cytotoxic granules (13). In the case reported here, the transcriptomic profile was compatible with both NK and a NK/T-cell population, the latter known to exhibit anti-leukemia activity (14, 15). The deficient function of the invariant NK/T cells in CML and their restoration on TKI therapy has previously been reported (16, 17). The role of NK cells in CML immune surveillance has also been suggested in patients remaining in deep molecular response after TKI discontinuation (11).

We then analyzed genes expressed in NK cell clusters among NK-T lymphocyte population in single-cell transcriptome, while the patient was in molecular recurrence and without TKI therapy but without hematological relapse. This NK cell cluster was positive for NKG7 expression and cells highly expressed effectors of cytotoxicity genes. Granulysin is an antimicrobial peptide (AMP) highly active against cancer cells such as melanoma (18). Cathepsin W (lymphopain) is a papain-like cysteine protease of unknown function that is specifically expressed in NK cells and to a lesser extent in cytotoxic T cells (CTL), and its expression in NK-92 (NK cell model) is linked to the cytotoxicity observed against K562 (19). Granzyme H is well-known cytotoxic effector of NK cells (20). NKG7+ positive NK cell cluster also highly expressed FGFBP2-secreted protein that is known to be a marker of the cytotoxicity of lymphocytes (21).

These molecular findings could also be linked to the immuno-molecular data obtained from the whole transcriptome analyses. Indeed, transcriptome experiments showed that the IL21R expression (receptor to interleukin 21) followed the BCR::ABL^-IS^ quantification analyses during TKI interruption period. IL-21 has been shown to participate to the NK cell expansion of K562 cell line engineered to express membrane-bound IL-21 feeder in combination with IL-2 (22, 23).

S1P (5) receptor found in NK cell network is also known to be essential for human NK cell trafficking (24, 25). Another interesting receptor expression was that of GPR56, which is an inhibitory receptor of NK cells, negatively regulating effector functions such as production of inflammatory cytokines and cytolytic proteins, degranulation, and target cell killing through association with the tetraspanin CD81 (26).

Overall, the molecular analyses reported during the long-term TKI discontinuation in this patient allowed to detect major modulation of genes involved with NK cell activity. Interestingly, several genes that we identified to be part of the immunological pathway seemed to be activated with a successful TFR after TKI discontinuation (10, 27–29). Importantly we have identified for the first time NKG7 expression in single cell transcriptome from CML PBMC, this gene being a major regulator of granule exocytosis in cancer (30). Its expression deserves further evaluation in patients with CML.

## Data availability statement

The datasets presented in this study can be found in online repositories. The names of the repository/repositories and accession number(s) can be found in the article/**Supplementary Material**.

## Ethics statement

The studies involving human participants were reviewed and approved by INSERM ethical committee. The patients/participants provided their written informed consent to participate in this study. Written informed consent was obtained from the patients/participants for the publication of this case report.

## Author contributions

JI, PM, NO and DC: Performed molecular Single cell experiments; CD: Bioinformatics analyses; XF performed BCR-ABL quantification experiments, JF, AB-G: validated BCR-ABL data, AT, AB-G: Clinical follow-up, plan experiments, JI, PM, CD, AB-G, AT: wrote the paper. All authors contributed to the article and approved the submitted version
